# Investigation of intracellular signals generated by γ-interferon and IL-4 leading to the induction of class II antigen expression

**DOI:** 10.1155/S096293519300047X

**Published:** 1993

**Authors:** S. Vassiliadis, N. Kyrpides, D. Stravopodis, M. Grigoriou, I. Athanassakis, J. Papamatheakis

**Affiliations:** 1 Institute of Molecular Biology and Biotechnology (IMBB) P.O. Box 1527 Crete Heraklion 711-10 Greece; 2 Dept of Biology University of Crete P.O. Box 1470 Crete Heraklion 711-10 Greece

## Abstract

Signal transduction plays a vital role in cellular behaviour as cells respond to various stimuli in different ways and utilize diverse pathways for accomplishing their task. Determination of the pathway followed by various cytokines can be achieved using specific inhibitors which include theophylline (TPH), TMB-8 and W7 that hinder calmodulin binding to Ca^2+^; sphingosine (SPH), H7 and staurosporine that inhibit protein kinase C (PKC) activation; and mevalonate (MEV) or the anti-p21^ras^ antibody which block G-proteins. This study shows that the immunologically important class II antigens in human cells are up-regulated predominately via the same pathway after gamma-interferon (γ-IFN) treatment, whereas murine cells are activated by other signalling routes. Thus, the calcium/calmodulin (Ca^2+^/Cam) pathway is preferentially selected for human cells whereas the PKC pathway is more often chosen for murine cells. These findings are firmly supported by other reports and show, in addition, a unique action exerted by γ-IFN, since IL-4, another inducer of class II antigen expression, uses different pathways. This diversity of activation reveals the existence of a previously unknown complicated network of intracellular interactions able to regulate the same phenotype or cellular event. As major histocompatibility complex antigens (MHC) or human leukocyte antigens (HLA), are important in immune recognition and response, the results show that for human cells a more coherent method of HLA-DR antigen induction is followed after γ-IFN administration, as calcium participation seems to be the first step in signal transduction. The same T-cell derived lymphokine, however, follows a totally different route when applied to murine cells.


Mediators of Inflammation 2, 343–348 (1993)

### Investigation of intracellular signals generated by γ-interferon and IL-4 leading to the induction of class II antigen expression

S. Vassiliadis,[1,CA] N. Kyrpides,[1,2] D. Stravopodis,[1,2] M. Grigoriou,[1,2] I. Athanassakis[2] and J. Papamatheakis[1,2]

[1] Institute of Molecular Biology and Biotechnology (IMBB), P.O. Box 1527, Heraklion 711-10, Crete, Greece;
[2] Dept of Biology, University of Crete, P.O. Box 1470, Heraklion 711-10, Crete, Greece

[CA] Corresponding Author

**SIGNAL transduction plays a vital role in cellular behaviour as cells respond to various stimuli in different ways and utilize diverse pathways for accomplishing their task. Determination of the pathway followed by various cytokines can be achieved using specific inhibitors which include theophylline (TPH), TMB-8 and W7 that hinder calmodulin binding to $Ca^{2+}$; sphingosine (SPH), H7 and staurosporine that inhibit protein kinase C (PKC) activation; and mevalonate (MEV) or the anti-p21$^{ras}$ antibody which block G-proteins. This study shows that the immunologically important class II antigens in human cells are up-regulated predominately via the same pathway after gamma-interferon (γ-IFN) treatment, whereas murine cells are activated by other signalling routes. Thus, the calcium/calmodulin ($Ca^{2+}$/Cam) pathway is preferentially selected for human cells whereas the PKC pathway is more often chosen for murine cells. These findings are firmly supported by other reports and show, in addition, a unique action exerted by γ-IFN, since IL-4, another inducer of class II antigen expression, uses different pathways. This diversity of activation reveals the existence of a previously unknown complicated network of intracellular interactions able to regulate the same phenotype or cellular event. As major histocompatibility complex antigens (MHC) or human leukocyte antigens (HLA), are important in immune recognition and response, the results show that for human cells a more coherent method of HLA-DR antigen induction is followed after γ-IFN administration, as calcium participation seems to be the first step in signal transduction. The same T-cell derived lymphokine, however, follows a totally different route when applied to murine cells.**

**Key words:** Class II antigens, γ-IFN, IL-4, Pathways followed, Signal transduction

### Introduction

Signal transduction is the term used for the series of stimuli occurring almost immediately after administration of a factor that will eventually lead cells to a mature and/or functional state. The level of activation may vary according to the transducer employed. Some agents affect cellular function by simply binding to specific surface receptors, with subsequent molecular activation of certain proteins; whereas other agents trigger a cascade of intracellular interactions that activate genes and their products in order to promote a distinct phenotype or to acquire a specific function not previously present.[1] Thus, cell cycle kinetics, or cellular, intracellular and nuclear behaviour can be manipulated by appropriate treatment(s).

Class II antigens can be activated by many naturally occurring substances—one of the best known being γ-IFN.[1–5] Previous studies have shown that γ-IFN exerts its action via different pathways depending on the cellular system examined and the type of gene under investigation.[5–8] Since immune recognition and response depend directly on class II antigens,[9] the authors examined how this agent up-regulates major histocompatibility complex (MHC)-Ia or human leukocyte antigen (HLA)-DR antigen expression in various cell systems. These groups of genes were chosen as they have been widely studied in murine and human cells respectively. The three most common pathways were examined, that is the $Ca^{2+}$/Cam[6,8] the PKC[3,10] and the G-protein system.[11–13]

When the $Ca^{2+}$/Cam second messenger pathway leads to up-regulation of class II surface antigens on various cells and/or cell lines,[6,8] this signalling mode may be interrupted by administering certain drugs, such as theophylline (TPH), W7 and TMB-8 that are known to be potent Cam inhibitors.[6,8] TPH also affects cAMP regulation, thus making cAMP an additional second messen-

© 1993 Rapid Communications of Oxford Ltd ger. However, such action appears to be $Ca^{2+}$/Cam dependent.[6]

PKC, when activated, leads to up-regulation of class II antigens via indirect means (other gene activation) as it is generally insufficient by itself to modulate HLA-DR expression.[3,7,10] Documented inhibitors of this pathway are sphingosine (SPH), H7 and staurosporine.[3,5,7]

The G-protein system participates as a signal transducer in a multiplicity of ways. One of them involves the product of the *ras* proto-oncogene, p21$^{ras}$.[2,13] It has been shown that induction of p21$^{ras}$ by γ-IFN or 5-azacytidine (5-AzaC) correlates with class II antigen expression in murine trophoblasts[2] and in HL-60 cells.[13] Inhibitors of such action are the anti-p21$^{ras}$ antibody[13] and mevalonate (MEV; which is contrary to results found in Ref. 11).

Although each of the above inhibitors acts on, and is specific for, a different part of its pathway, it is becoming clear that there is possible 'cross-talking' between the pathways, an issue currently being explored in many laboratories.[14] Each pathway is, in itself, a complex and often difficult to follow network of interactions involving numerous byproducts. Therefore, this work is based on the assumption that the inhibitors used are specific for their corresponding pathways and hence class II antigen expression is indeed the final product of the various reactions. As this type of investigation is tedious, the pathway(s) difficult to follow and the results mixed, well characterized and well documented inhibitors (see Methods and Results) were used.

This study shows that γ-IFN exhibits a more stable behaviour in inducing HLA-DR antigens on human HeLa and HL-60 cells, as $Ca^{2+}$/Cam appears to be the major pathway followed. There is evidence that this agent also follows the same pathway for human U937 cells[6] and normal monocytes.[5] In the murine system, however, the situation is totally different as γ-IFN follows either the $Ca^{2+}$/Cam or the PKC pathway for inducing MHC-Ia.

An important aspect in the investigation of signal transduction is the multiplicity of ways a factor may cause the activation of certain genes. Different transducers may influence the expression of the same genes by following totally different and independent pathways. For instance, it has been shown that interleukin-4 (IL-4), which also up-regulates MHC and HLA antigens,[15] either shares or selects distinct pathways for class II antigen expression. Thus, IL-4 induces HLA-DR expression via the G-protein system on HL-60 and normal monocytic cells[13] whereas this present work shows that it mimics γ-IFN when inducing MHC-Ia antigens on the WEHI-3 murine macrophage-like cells.

### Materials and Methods

*Cells and cell cultures:* The human epithelial-like cell line HeLa and the promyelocytic leukaemia HL-60 were purchased from ATCC (Rockville, MD, USA). The murine macrophage-like cell line WEHI-3 and the murine pre-B line 70Z were also purchased from ATCC. For control purposes the human pre-B cell line 6.1.6 (ATCC) was also used.

HL-60, 70Z and 6.1.6 cells were grown in RPMI (Gibco, NY, USA) supplemented with 10% FCS (Seralab, Sussex, UK) and maintained in a humidified atmosphere of 5% $CO_2$ at 37°C. The growth of HeLa and WEHI-3 cell lines was supported in Dulbecco's modified Eagle's medium (DMEM, Gibco) using the same conditions as described above.

Normal human monocytes were purified (> 95%) from human blood by Percoll's density gradient centrifugation and adhesion to plastic dishes. Cells were cultured in RPMI 1640 medium supplemented with 10% FCS at 37°C in 5% $CO_2$.

*Inducers of class II antigens and induction protocols:* Class II surface antigens in both murine and human cells were up-regulated by recombinant γ-IFN (Holland Biotech, Leiden, Holland, and Genzyme, Boston, USA for mouse and human IFN respectively) and recombinant human and murine IL-4 (Genzyme). Human cells were treated with human factor preparations, whereas murine cell lines were cultured with their corresponding species-specific interleukins.

Doses of γ-IFN and IL-4, for the up-regulation of class II antigens, were selected as described previously.[8,15] Briefly, dose–response curves were plotted as a function of time and concentrations were chosen at levels before reaching plateau values (data not shown). Thus, all cells were treated for 48 h, unless indicated otherwise, with 100 U/ml of rγ-IFN, a dose given at the beginning of the culture. IL-4 was administered at a concentration of 200 U/ml for HL-60 and 100 U/ml for HeLa and WEHI-3 cells. As reported previously, a single dose of IL-4 does not suffice for class II induction as it is rapidly used by the cells.[15] Therefore, an initial dose was given at the beginning of the culture and a second boost 24 h before collecting the cells.

*Chemicals/signal transduction inhibitors:* Theophylline (TPH), sphingosine (SPH) and mevalovate (MEV) were all purchased from Sigma chemicals (St Louis, MO, USA). TPH is known to block the $Ca^{2+}$/Cam pathway at a concentration of 0.15 mg/ml.[8] Sphingosine, a PKC inhibitor, was used at a final concentration of 50 nM,[5] whereas MEV, known to block G-protein activation (laboratory results), was added at a concentration of 0.5 mg/ml. W7 (Sigma) a Cam inhibitor (15 μM), staurosporine (UBI, Lake

Placid, NY) a PKC inhibitor (50 nM) and anti-p21$^{ras}$ antibody (Oncogene Sci., Manhasset, NY) a G-protein inhibitor (1 μg/ml) were also used and gave identical results as their reported counterparts theophylline, sphingosine and mevalonate. Where not referenced, doses were selected after appropriate toxicity tests (cell viability over 90% as assessed by Trypan dye exclusion and ability to incorporate $^3$H-TdR in proliferation assays). All inhibitors were diluted as instructed in the Merck manual as they are not water soluble. The tyrosine kinase (TK) inhibitor genistein (UBI) at 30 μg/ml was used as an additional control and potential inhibitor of class II antigens. (See Discussion and legend to Table 1).

*Class II antigen assessment—Dynabead binding and Northern analysis:* MHC Ia and HLA-DR antigens were determined by specific binding of DynaBeads (Dynal, Norway) and Northern analysis. For visual detection murine WEHI-3 cells were first reacted with an anti-IA$^d$ monoclonal antibody (Becton Dickinson) as these cell derived from a BALB/c mouse according to standard procedures.[2] 70Z cells, a (b × d) hybrid, were also reacted with the same IA$^d$ antibody. Then beads coated with an anti-mouse IgG were added according to the manufacturer's instructions. Human cells (HeLa and HL-60) were incubated directly at 4°C with beads coated with anti-DR antigens as published previously.[13] Specific binding (rosetting) was monitored using an Olympus microscope.

Northern analysis was carried out as described previously.[14] The probes employed were the PDRH2 fragment (DR α)[15] for the human cells and Eα for the murine counterparts as reported previously.[2]

### Results

Results obtained from Dynabead binding experiments, showing the percentage of class II antigen expression in human and murine cells after treatment with the various agents, are shown in Table 1. All the inhibitors were used at non-toxic concentrations (see Methods) as assessed by cell viability tests (over 90%) and incorporation of $^3$H-TdR (data not shown). Treatment of all cells with either TPH, SPH or MEV only, did not affect class II antigen expression.

The tyrosine kinase (TK) inhibitor, genistein, was used as an additional and also potential inhibitor of class II antigen expression. However, it was unable to block the antigen expression (data not shown).

In human pre-B 6.1.6 cells neither γ-IFN nor IL-4 were able to induce class II antigen expression (data not shown). However, high doses of human IL-6 or the combination of LPS + DMSO can be used successfully as inducers of HLA-DR (data not shown).

**Table 1.** Pathways followed during the induction of class II antigens in human and mouse cells after treatment with recombinant γ-IFN and IL-4

| Treatment | Percentage of class II antigen expression* | | | |
|---|---|---|---|---|
| | Human cells | | Murine cells | |
| | HeLa | HL-60 | WEHI-3 | 70Z |
| None | 8 ± 2 | 8 ± 2 | 8 ± 2 | 18 ± 1 |
| TPH | 4 ± 1 | 7 ± 2 | 10 ± 1 | 21 ± 3 |
| SPH | 5 ± 1 | 8 ± 1 | 10 ± 2 | 20 ± 5 |
| MEV | 6 ± 1 | 10 ± 2 | 11 ± 1 | 20 ± 1 |
| γ-IFN only | 25 ± 3 | 45 ± 2 | 52 ± 5 | 20 ± 2 |
| γ-IFN + TPH | 8 ± 2** | 16 ± 3** | 48 ± 4 | 19 ± 2 |
| γ-IFN + SPH | 20 ± 2 | 40 ± 5 | 20 ± 2** | 22 ± 3 |
| γ-IFN + MEV | 21 ± 3 | 40 ± 4 | 48 ± 4 | 22 ± 2 |
| IL-4 only | 11 ± 1 | 35 ± 2 | 40 ± 2 | 45 ± 3 |
| IL-4 + TPH | 10 ± 1 | 33 ± 3 | 35 ± 3 | 53 ± 7 |
| IL-4 + SPH | 12 ± 2 | 33 ± 2 | 7 ± 2** | 26 ± 2** |
| IL-4 + MEV | 10 ± 2 | 12 ± 2** | 38 ± 3 | 40 ± 6 |

* As assessed by specific Dynabead binding as described in the Methods. The experiments have been performed at least four times and the numbers represent mean values ± S.D. According to Student's *t*-test, all values marked with an asterisk are statistically significant compared with their corresponding induced percentages.
** Indicates suppressive agent and pathway of choice when compared with appropriate controls.

In the human cell lines, HeLa and HL-60, treatment with γ-IFN alone, caused 3- and 6-fold increases, respectively, in class II antigen expression. In control experiments γ-IFN was unable to induce these antigens on human pre-B 6.1.6 cells (data not shown). When SPH or MEV were included with γ-IFN in the assay mixture, antigen expression was increased by a similar amount to that with γ-IFN alone. However, the inclusion of TPH with γ-IFN caused a significant inhibition of class II antigen expression, and results were similar to those of untreated cells. Figure 1 shows the results of Northern blot analysis for the reaction of IFN and TPH with HeLa cells. The reduced quantity of antigen expression after reaction with IFN + TPH, compared with IFN alone, can be seen.

In contrast to γ-IFN, in human cells, IL-4 caused a four-fold increase of class II antigen expression only in HL-60 cells. This increase was abolished by the inclusion of MEV in the assay mixture (Table 1).

In the murine cell line WEHI-3, IFN and IL-4 caused seven- and five-fold increases, respectively, in class II antigen expression (Table 1). However, only IL-4 increased antigen expression in 70Z cells. In both cell lines, the combination of either IFN + SPH or IL-4 + SPH significantly reduced

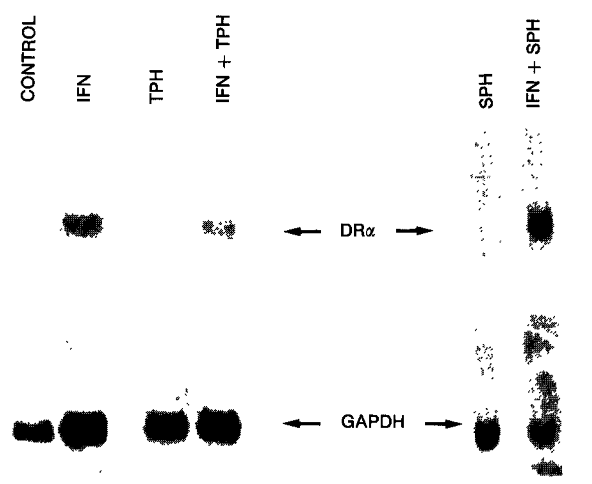

FIG 1. Northern analysis of human HeLa cells showing that class II antigen induction at the mRNA level takes place via the $Ca^{2+}$/Cam pathway after treatment with γ-IFN for 48 h. Theophylline (TPH) blocks the expression of HLA-DR, whereas sphingosine (SPH) has no effect. Note that although more RNA (GAPDH quantity control) has been loaded in the IFN lane, spectrophotometric analysis has shown that the IFN induction is significant.

the increases in antigen expression; whereas when either TPH or MEV were included in the assay mixtures, the percentage of antigen expression was similar to that with either IFN or IL-4 alone.

Figure 2 shows the results of Northern blot analysis for the reaction of IL-4 and SPH with WEHI-3 cells. The reduced antigen expression after reaction with IL-4 + SPH, compared with IL-4 alone, can be seen.

### Discussion

As immune recognition and response depend directly on class II antigen expression, and MHC-Ia and HLA-DR belong to the category of inducible genes, the signal transducing pathway followed by γ-IFN in the up-regulation of class II surface antigens on human and murine cells was examined. Many cellular systems have been studied after induction with γ-IFN or other cytokines and have yielded evidence of a diverse pattern of routes that are sometimes unique and sometimes intra-related, as a considerable amount of 'cross-talking' between the pathways exists. In this work, human and murine cell systems were examined to determine whether γ-IFN follows a specific pathway. By treatment human HeLa and HL-60 as well as mouse WEHI-3 macrophage-like cells the inductive capacity of this agent in up-regulating class II antigens was demonstrated (Table 1). In the presence of γ-IFN class II antigen expression increased up to six-fold in these cell lines (Table 1). By using chemicals that are specific inhibitors of certain pathways followed in signal transduction, the path followed by γ-IFN was deduced. The results in Table 1 show that γ-IFN alone, or in combination with either SPH or MEV, caused similar upregulation of class II antigen expression in both HeLa and HL-60 cells. However, the presence of TPH (a specific inhibitor of the $Ca^{2+}$/Cam pathway) with γ-IFN significantly reduced class II antigen expression. The results therefore indicate that, in these human cells, γ-IFN causes up-regulation via the $Ca^{2+}$/Cam pathway. In addition, when human normal monocytes are used as the cellular system (a direct analogue to the normal situation) it is found that γ-IFN also follows the $Ca^{2+}$/Cam pathway, as TPH blocks up-regulation of HLA-DR surface antigens (25 ± 2% in control cells *vs* 60 ± 4% after IFN treatment and 18 ± 3% in the IFN + TPH combination). These results are presented as a summary in Table 2. This observation is also confirmed by other indirect studies where PKC activators failed to induce class II antigens on these cells.[5]

In contrast to γ-IFN, IL-4 only affected antigen expression in HL-60 cells. The results in Table 1 suggest that IL-4 induces class II antigens via the G-protein system, as only IL-4 + MEV (an inhibitor of the G-protein system) significantly reduced antigen expression. This data confirms previously reported findings using human normal monocytes where the participation of the G-protein system was detected using an anti-$p21^{ras}$ antibody.[13]

Treatment of cells with anti-$p21^{ras}$ antibody does not, however, affect class II antigen expression induced by γ-IFN (60 ± 4% after IFN treatment *vs* 53 ± 5% in the IFN + $p21^{ras}$ antibody combination). From other literature sources it is known that in the U937 cell line $Ca^{2+}$ influx is also involved,[6] whereas for the more mature THP-1 cell line, it has been shown that PKC interferes as a late-acting mechanism in DR induction which is not sufficient

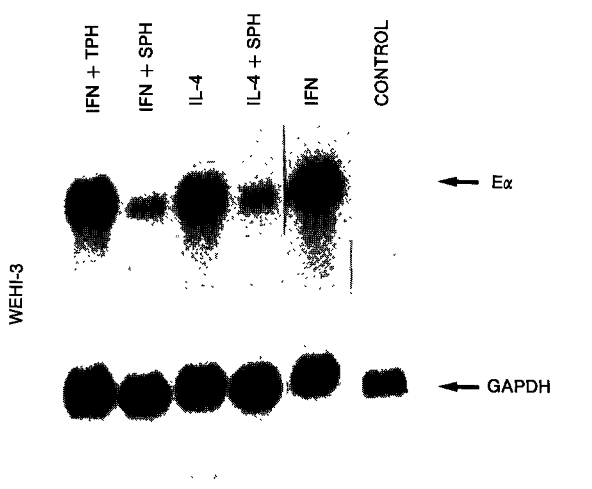

FIG 2. Northern analysis of mouse WEHI-3 cells demonstrating (a) the MHC-Ia inductive capacity of γ-IFN and IL-4 at the mRNA level, and (b) the pathways followed in up-regulating class II antigens (Eα). The actions of both γ-IFN and IL-4 can be inhibited by the PKC inhibitor sphingosine (SPH). Theophylline (TPH), which blocks the $Ca^{2+}$/Cam pathway, does not have any effect.

**Table 2.** Summary of signal transduction pathways followed by γ-IFN in up-regulating class II antigen expression in human and mouse cells

| Cell type | Pathway of class II antigen induction after γ-IFN treatment | Inhibitors*/ interpretation |
|---|---|---|
| Human | | |
| HeLa | $Ca^{2+}$/Cam | TPH (This work) |
| HL-60 | $Ca^{2+}$/Cam | TPH (This work and Reference 8) |
| U937 | $Ca^{2+}$ influx | TMB-8 and W7[6] |
| THP-1 | Early: unknown | ? |
| | Late: PKC | H7[5,7] |
| Normal monocytes | $Ca^{2+}$/Cam | a) PKC activators fail to induce DR[5] |
| | | b) TPH (Unpublished**; see Results) |
| Murine | | |
| WEHI-3 | PKC | SPH (This work) |
| Bone marrow macrophages | PKC-independent | SPH, staurosporine do not inhibit[4] |
| Peritoneal macrophages | $Na^+/H^+$ and/or PKC | Reviewed in Reference 3 |
| Blood macrophages | PKC | a) PKC activation[1,3] |
| | | b) SPH (Unpublished**) |
| Placenta | $Ca^{2+}$/Cam and PKC | TPH and SPH (Unpublished**) |
| Astrocytes (rat) | PKC and $Na^+$ entry | a) H7 inhibits $Na^+$ and then MHC-Ia[3] |
| | | b) H7 directly inhibits PKC[3] |

* TPH, W7 and TMB-8 are $Ca^{2+}$/Cam inhibitors; SPH, H7 and staurosporine block PKC activation.
** Unpublished results from the authors' laboratory.

by itself for such up-regulation.[5] It is probable that the early signal transduced, although not yet elucidated, is $Ca^{2+}$-dependent. Thus, in different cellular systems having a common origin (myeloid in different stages of maturation) plus an epithelial-like cell line, HeLa, class II antigen expression can be induced by γ-IFN via the $Ca^{2+}$/Cam pathway. Such a finding shows a selective route not reported for any other inducer. Thus, for the human system, there is a coherent mode of action followed by γ-IFN thus making the $Ca^{2+}$/Cam pathway unique in the sense that it is either distinct from the other pathways or it represents the first signal for the cascade of interactions to follow. Furthermore, despite the variability of the inhibitors used (TPH *vs* W7 and TMB-8), the results obtained are the same.

The murine cellular system, however, exhibits totally different behaviour as other intracellular signals regulate induction of MHC-Ia. In WEHI-3 cells γ-IFN induces a seven-fold increase in class II antigen expression. It is considered that this induction occurs via the PKC pathway as it can be inhibited by SPH and staurosporine, whereas TPH and MEV do not affect the expression (Table 1). IL-4, which also potently induces MHC-Ia (five-fold), mimics the action of γ-IFN by following the same PKC route. The only difference in the inductive capacity of the two interleukins is the double dose of IL-4 added to the cells. The first dose at the beginning of the culture is not sufficient to up-regulate class II antigens at 48 h (data not shown) as the factor is rapidly consumed (as occurs with human cells[15]). The inductions caused by both γ-IFN and IL-4, however, are inhibited by SPH, a finding also confirmed by Northern analysis (Fig. 2).

Murine pre-B cells (70Z) are induced to express MHC-Ia antigens only after IL-4 treatment. This up-regulation also follows the PKC route as only SPH inhibits this event (Table 1). In contrast to HLA-DR, normal bone marrow macrophages, peritoneal and blood macrophages up-regulate MHC-Ia after γ-IFN treatment in a manner which is not dependent on the $Ca^{2+}$/Cam pathway (Table 2). In these systems $Na^+$ appears to be an important parameter in signal transduction.[3] For murine normal blood macrophages the authors found that SPH blocks induction (data not shown) confirming other reports showing that γ-IFN directly activates the PKC pathway.[4] When working with murine placenta cells and γ-IFN, it is concluded that both $Ca^{2+}$/Cam and PKC are involved in signal transduction as up-regulation of class II antigens is abolished by inhibitors of either pathway (Table 2 and data not shown). This observation suggests the existence of common or shared pathways followed for the accomplishment of a cellular event. However, the possibility cannot be excluded that all pathways may interact at a certain level during activation and that such interaction compensates for other weaknesses of the system during gene activation. Evidence for such pathway interaction is obtained from the reactions of many phosphatases (such as protein phosphatase 2B, PP2B) that are able to reverse the action of PKC and are also $Ca^{2+}$/Cam dependent.[16,17] Therefore, Cam antagonists such as EGTA or TPH may inhibit the action of such phosphatases and allow the action of PKC to continue. Alternatively, as in the case of PP2B, the precursor protein phosphatase molecules 1 and 2A (conversion of PP1 to PP2A) may themselves inhibit PKC[18] and therefore a Cam antagonist is insufficient to act later at the PP2B level. These findings, although illuminating, create an endless circle of arguments and make the delineation of the signalling pathways a difficult task. In the cytoplasmic domain of a cell, therefore, an infinite number of interactions may dictate the routes of gene activation. Some of these routes have already been published either as models or facts.[14]

Although the tyrosine kinase (TK) inhibitor genistein had no effect on any of the cellular systems studied in this work, the participation of the TK pathway is discussed here as recent publications suggest potential roles for TK in the up-regulation of class II antigens and control of lymphocyte activation. As the antigen receptors on B- and T-cells (BcR and TcR) are linked to the PKC pathway via phospholipase C (PLC) and diacylglycerol (DAG),[19] studies have been undertaken to show the concomitant involvement of G proteins/p21$^{ras}$ and TK in this type of activation. Although evidence exists for such actions,[19] there is also a great deal of debate concerning the various isoforms of PLC and their net, as well as a questionable involvement in other pathways. Furthermore, G-proteins/p21$^{ras}$ appear to be an important mediator during T-cell activation, for example, it may control the IL-2 gene promoter.[20] Also in haemopoietic cells, *ras* activation has been found to occur in response to several growth factors (IL-2, IL-3, GM–CSF and steel factor SFL) thought to be linked to TKs.[21] However, the mechanisms of *ras* activation are not yet fully understood. Finally, it has been shown that tyrosine phosphorylation controls several other cellular functions such as the responses stated above[21] as well as the γ-IFN-induced HLA-DR expression in the human glioblastoma cell line T98G.[22] In this latter work, γ-IFN-induced class II antigen expression will prevail provided that TK phosphorylation does not take place. However, in this case the pathway followed by γ-IFN has not been studied.

In conclusion, this work sheds some light on a poorly understood field of study and shows that a well characterized interleukin, γ-IFN, follows distinct pathways for the up-regulation of class II antigens in human and mouse cells. After γ-IFN administration, human cells are stimulated preferentially through the $Ca^{2+}$/Cam pathway whereas murine cells exhibit a preference for the PKC route which appears to be coupled to other intracellular events and shares common routes with the $Ca^{2+}$/Cam system. The conclusions of this work, that stem from the study of only well known indicator cell lines examined here, are strongly supported by other studies performed on a variety of cell types as presented in Table 2.

### References

1. Adams DO, Hamilton TA. Molecular transductional mechanisms by which IFN-γ and other signals regulate macrophage development. *Immunol Rev* 1987; **97**: 5–27.
2. Athanassakis-Vassiliadis I, Vassiliadis S, Papamatheakis J. Common mechanisms govern the expression of p21*ras* and class II MHC antigens in the murine placenta. *J Reprod Immunol* 1991; **21**: 149–161.
3. Benveniste ET, Vidovic M, Panek RB, Norris JG, Reddy AT, Benos DJ. Interferon-γ-induced astrocyte class II MHC gene expression is associated with both PKC and $Na^+$ entry. *J Biol Chem* 1991; **266**: 18119–18126.
4. Celada A, Maki RA. γ-IFN induces the expression of the genes for MHC class II I-Aβ and TNF through a PKC-independent pathway. *J Immunol* 1991; **146**: 114–121.
5. Gumina RJ, Freire-Moar J, DeYoung L, Webb DR, Devens BJ. Transduction of the γ-IFN signal for HLA-DR expression in the promonocytic line THP-1 involves a late-acting PKC activity. *Cell Immunol* 1991; **138**: 256–279.
6. Ina Y, Koide Y, Nezu N, Yoshida TO. Regulation of HLA class II antigen expression: intracellular signaling molecules responsible for the regulation by IFN and cross-linking of Fc receptors in HL–60 cells. *J Immunol* 1987; **139**: 1711–1717.
7. Mao C, Merlin G, Aguet M. Differential regulation of the human γ-IFN receptor expression in Raji and IM9 lymphoblastoid cells versus THP-1 monocytic cells by γ-IFN and PMA. *J Immunol* 1990; **144**: 4688–4693.
8. Vassiliadis S, Guilbert LJ. Immune interferon induces early and late events in differentiation of HL-60 cells that are part of the same maturation program. *Exp Hematol* 1991; **19**: 250–256.
9. Yem AW, Darmley MJ. Modulation of Ia-like antigen expression and antigen presenting activity of human monocytes by endotoxin and zymosan A. *J Immunol* 1981; **127**: 2245–2252.
10. Yasuda I, Kishimoto A, Tanaka S, Tominaga M, Sakurai A, Nishizuka Y. A synthetic peptide substrate for selective assay of PKC. *Bioch Bioph Res Comm* 1990; **166**: 1220–1227.
11. Schafer WR, Kim R, Sterne R, Thorner J, Kim SH, Rine J. Genetic and pharmacological suppression of oncogenic mutations in *ras* genes of yeast and humans. *Science* 1989; **245**: 379–381.
12. Stone JC, Blanchard RA. Genetic definition of *ras* effector elements. *Molec Cell Biol* 1991; **11**: 6158–6165.
13. Vassiliadis S, Papamatheakis J. The p21$^{ras}$ protein as an intermediate signaling molecule in the IL-4-induced HLA-DR expression on normal and leukemic human myeloid cells. *Cell Immunol* 1992; **142**: 426–433.
14. The October issue of TIBS. Signal transduction: crosstalk. *TIBS* 1992; **17**: 367–443.
15. Vassiliadis S, Kyrpides N, Papamatheakis J. The role of IL-4 in human myeloid leukemia: stimulation of RNA synthesis and transduction of differentiation signals through an IL-4 receptor leads to functional and HLA positive HL-60 cells. *Leuk Lymphoma* 1992; **7**: 235–242.
16. Coehn P, Klumpp S, Schelling DL. An important procedure for identifying and quantitating protein phosphatases in mammalian tissue. *FEBS Letters* 1989; **250**: 596–600.
17. Redpath NT, Proud CG. The tumour promoter okadic acid inhibits reticulocyte-lysate protein synthesis by increasing the net phosphorylation of elongation factor 2. *Biochem J* 1989; **262**: 69–75.
18. Haystead TAJ, Sim ATR, Carling D, Honnor RC, Tsukitani Y, Coehn P, Hardie DCT. Effects of the tumour promoter okadaic acid on intracellular protein phosphorylation and metabolism. *Nature* 1989; **337**: 78–81.
19. Harnett M, Rigley K. The role of G-proteins versus protein tyrosine kinases in the regulation of lymphocyte activation. *Immun Today* 1992; **13**: 482–486.
20. Rayter SI, Woodrow M, Lucas SC, Cantrell DA, Downward J. p21ras mediates control of IL-2 gene promoter function in T cell activation. *EMBO J* 1992; **11**: 4549–4556.
21. Duronio V, Clark-Lewis I, Federsppiel B, Wieler JS, Schrader JW. Tyrosine phosphorylation of receptor β subunits and common substrates in response to IL-3 and GM–CSF. *J Biol Chem* 1992; **267**: 21856–21863.
22. Ryu K, Koide Y, Yamashita Y, Yoshida TO. Inhibition of tyrosine phosphorylation prevents IFN-γ-induced HLA-DR molecule expression. *J Immunol* 1993; **150**: 1253–1262.

ACKNOWLEDGEMENT. This work was partially supported by IMBB funding.

**Received 17 May 1993; accepted in revised form 5 July 1993**

## References

[PDF_00608] Adams D. O., Hamilton T. A. (1987). Molecular transductional mechanisms by which IFN gamma and other signals regulate macrophage development.. Immunol Rev.

[PDF_00611] Athanassakis-Vassiliadis I., Vassiliadis S., Papamatheakis J. (1992). Common mechanisms govern the expression of p21ras and class II MHC antigens in the murine placenta.. J Reprod Immunol.

[PDF_00614] Benveniste E. N., Vidovic M., Panek R. B., Norris J. G., Reddy A. T., Benos D. J. (1991). Interferon-gamma-induced astrocyte class II major histocompatibility complex gene expression is associated with both protein kinase C activation and Na+ entry.. J Biol Chem.

[PDF_00617] Celada A., Maki R. A. (1991). IFN-gamma induces the expression of the genes for MHC class II I-A beta and tumor necrosis factor through a protein kinase C-independent pathway.. J Immunol.

[PDF_00654] Cohen P., Klumpp S., Schelling D. L. (1989). An improved procedure for identifying and quantitating protein phosphatases in mammalian tissues.. FEBS Lett.

[PDF_00668] Duronio V., Clark-Lewis I., Federsppiel B., Wieler J. S., Schrader J. W. (1992). Tyrosine phosphorylation of receptor beta subunits and common substrates in response to interleukin-3 and granulocyte-macrophage colony-stimulating factor.. J Biol Chem.

[PDF_00620] Gumina R. J., Freire-Moar J., DeYoung L., Webb D. R., Devens B. H. (1991). Transduction of the IFN-gamma signal for HLA-DR expression in the promonocytic line THP-1 involves a late-acting PKC activity.. Cell Immunol.

[PDF_00663] Harnett M., Rigley K. (1992). The role of G-proteins versus protein tyrosine kinases in the regulation of lymphocyte activation.. Immunol Today.

[PDF_00660] Haystead T. A., Sim A. T., Carling D., Honnor R. C., Tsukitani Y., Cohen P., Hardie D. G. (1989). Effects of the tumour promoter okadaic acid on intracellular protein phosphorylation and metabolism.. Nature.

[PDF_00624] Ina Y., Koide Y., Nezu N., Yoshida T. O. (1987). Regulation of HLA class II antigen expression: intracellular signaling molecules responsible for the regulation by IFN-gamma and cross-linking of Fc receptors in HL-60 cells.. J Immunol.

[PDF_00665] Rayter S. I., Woodrow M., Lucas S. C., Cantrell D. A., Downward J. (1992). p21ras mediates control of IL-2 gene promoter function in T cell activation.. EMBO J.

[PDF_00657] Redpath N. T., Proud C. G. (1989). The tumour promoter okadaic acid inhibits reticulocyte-lysate protein synthesis by increasing the net phosphorylation of elongation factor 2.. Biochem J.

[PDF_00671] Ryu K., Koide Y., Yamashita Y., Yoshida T. O. (1993). Inhibition of tyrosine phosphorylation prevents IFN-gamma-induced HLA-DR molecule expression.. J Immunol.

[PDF_00640] Schafer W. R., Kim R., Sterne R., Thorner J., Kim S. H., Rine J. (1989). Genetic and pharmacological suppression of oncogenic mutations in ras genes of yeast and humans.. Science.

[PDF_00643] Stone J. C., Blanchard R. A. (1991). Genetic definition of ras effector elements.. Mol Cell Biol.

[PDF_00631] Vassiliadis S., Guilbert L. J. (1991). Immune interferon induces early and late events in differentiation of HL-60 cells that are part of the same maturation program.. Exp Hematol.

[PDF_00650] Vassiliadis S., Kyrpides N., Papamatheakis J. (1992). The role of IL-4 in human myeloid leukemia: stimulation of RNA synthesis and transduction of differentiation signals through an IL-4 receptor leads to functional and HLA positive HL-60 cells.. Leuk Lymphoma.

[PDF_00645] Vassiliadis S., Papamatheakis J. (1992). The p21ras protein as an intermediate signaling molecule in the IL-4-induced HLA-DR expression on normal and leukemic human myeloid cells.. Cell Immunol.

